# *Salmonella* Typhi From Blood Cultures in the Democratic Republic of the Congo: A 10-Year Surveillance

**DOI:** 10.1093/cid/ciy1116

**Published:** 2019-03-07

**Authors:** Bieke Tack, Marie-France Phoba, Sandra Van Puyvelde, Lisette M Kalonji, Liselotte Hardy, Barbara Barbé, Marianne A B Van der Sande, Elise Monsieurs, Stijn Deborggraeve, Octavie Lunguya, Jan Jacobs

**Affiliations:** 1 Department of Clinical Sciences, Institute of Tropical Medicine, Antwerp; 2 Department of Microbiology and Immunology, KU Leuven, Belgium; 3 Department of Microbiology, National Institute for Biomedical Research; 4 Department of Microbiology, University Teaching Hospital, Kinshasa, Democratic Republic of the Congo, Antwerp, Belgium; 5 Department of Biomedical Sciences, Institute of Tropical Medicine, Antwerp, Belgium; 6 Wellcome Trust Sanger Institute, Hinxton, United Kingdom; 7 Department of Public Health, Institute of Tropical Medicine, Antwerp, Belgium; 8 Julius Center for Health Sciences and Primary Care, Global Health Centre, Utrecht University, The Netherlands; 9 Royal Museum for Central Africa, Tervuren; 10 Department of Geography, University of Liège, Belgium

**Keywords:** typhoid fever, *Salmonella* Typhi, Democratic Republic of the Congo, surveillance

## Abstract

**Background:**

This study gives an overview of a decade (2007–2017) of hospital-based *Salmonella* Typhi bloodstream infection (BSI) surveillance in the Democratic Republic of the Congo (DRC), at 4 main sampling sites.

**Methods:**

Blood cultures were sampled in hospital-admitted patients with suspected BSI, according to standardized clinical indications. The results of the surveillance period 2015–2017 were compiled with those of previous surveillance periods (2007–2010 and 2011–2014). Whole genome sequencing of isolates with decreased ciprofloxacin susceptibility (DCS) was performed.

**Results:**

*Salmonella* Typhi was isolated in 1.4% (531/37 388) and 10.3% (531/5177) of suspected and culture-confirmed BSI episodes, respectively. *Salmonella* Typhi ranked first among the BSI pathogens in adults (n = 220), but was mostly (n = 301 [56.7%]) isolated from children, of which 72.1% (217/301) and 31.6% (95/301) were <10 years and <5 years old, respectively. Multidrug resistance (MDR), DCS, and combined MDR/DCS were found in 38.3% (n = 180), 24.5% (n = 115), and 11.9% (n = 56) of 470 first isolates, respectively. MDR and DCS rates had increased since 2007, but remained stable during 2015–2017 with no geographical clustering at the province level. Most (91/93 [97.8%]) DCS isolates sequenced belonged to Genotyphi genotype 2.5.1, and gyr S83 was the most frequent DCS mutation (76/93 [81.7%]). Infections occurred perennially, but increased during the rainy season.

**Conclusions:**

*Salmonella* Typhi was a frequent cause of BSI in adults and children in DRC, with high rates of antibiotic resistance. Sustainable surveillance and implementation of vaccination are compelling.

Typhoid fever is a poverty-related disease with a high disease burden in low- and middle-income countries [1, 2]. Its causative organism—*Salmonella enterica* subspecies *enterica* serovar Typhi—has been listed on the World Health Organization (WHO) priority list of antibiotic-resistant bacteria [3]. Recent studies have confirmed high incidence rates of typhoid fever in sub-Saharan Africa, which have incited vaccination efforts [4–6]. Central Africa has been estimated to face the worldwide highest incidence of typhoid fever [7]. As to the Democratic Republic of the Congo (DRC), the country’s poor infrastructure and health services (Supplementary Table 1) make it vulnerable to typhoid fever, and clinical and epidemiological observations have confirmed its frequency and impact [8, 9]. In the aftermath of the typhoid fever peritonitis outbreak in 2005 [8], the National Institute of Biomedical Research (INRB) in Kinshasa started, in collaboration with the Institute of Tropical Medicine (ITM) in Antwerp, Belgium, a microbiological surveillance in sentinel hospitals around the country with the initial objectives to investigate and confirm epidemic alerts of typhoid fever and to assess the antibiotic resistance of *Salmonella* Typhi [10, 11].

The present study reports, for the period 2007–2017 in DRC, the cumulative temporal, spatial, and age distributions of *Salmonella* Typhi blood culture isolates, their antimicrobial resistance patterns, and their genetic relatedness to the global *Salmonella* Typhi population.

## METHODS

### Microbiology Surveillance Network in DRC

Blood cultures were collected and processed free of charge in healthcare facilities in the capital, Kinshasa, and the Bas-Congo province (referral hospital of Kisantu) and later extended to sentinel hospitals in the Oriental Province (University Hospital Kisangani with affiliated hospitals and health centers) and the Equateur Province (referral hospital of Bwamanda). Over the years, this network was extended with other partners, and blood cultures were also added to the diagnostic tools of the outbreak investigation teams of INRB in collaboration with the Ministry of Health. Indications of blood culture sampling were, outside the neonatal period, a body temperature of ≥38.0°C or ≤35.5°C (axillary) or history of fever during the last 48 hours and/or (1) signs of severity such as hypotension, confusion, or increased respiratory rate; (2) suspicion of severe localized infections (pneumonia, meningitis, complicated urinary tract infection, osteomyelitis and arthritis, severe skin and soft tissue infections, gynecological and abdominal infections/peritonitis); or (3) suspicion of other severe infection: sepsis, typhoid fever, and severe malaria. Basic demographic and clinical data were recorded. In adults, blood cultures consisted of 2 × 10 mL of blood sampled in aerobic BACT/ALERT bottles (bioMérieux, Marcy-L’Etoile, France); for children (defined as ≤14 years old), 1–4 mL was sampled in pediatric BACT/ALERT bottles. Grown blood cultures were worked up for identification according to conventional biochemical methods and serotyping (for antibiotic susceptibility testing, see below). For reasons of integration in clinical care and capacity building, blood cultures were preferentially worked up in the participating referral hospitals. However, when no competent laboratory services were available, samples were transported to INRB for workup. Isolates were stored for in-depth reference testing, and the results of the blood culture isolates and their resistance patterns were registered. Coagulase-negative *Staphylococcus*, *Corynebacterium* species, *Micrococcus* species, *Cutibacterium* species, and *Lactobacillus* species were considered as environmental or skin contaminants; the other bacteria were classified as pathogens. The term “suspected bloodstream infection (BSI) episode” was used to indicate all blood cultures in a single patient sampled within a 2-week period according to the indications mentioned above. A BSI episode was considered as culture confirmed when a pathogen was isolated in at least 1 of the bottles. Supplementary Document 1 provides a detailed overview of the workflow, criteria, and definitions used in the sampling, and the processing and reporting of the blood cultures.

### Design of the Present Study

The blood culture isolates from the period January 2015–October 2017 were retrieved from storage at –80°C and batch-tested for identification and antibiotic susceptibility. To allow a complete 10-year retrospective view, a comprehensive database was compiled using the most recent data from 2015–2017 and 2 databases used for 2 previous *Salmonella* Typhi reports, comprising the periods April 2007–January 2011 (further referred to as “2007–2010”) [10] and January 2011–December 2014 (referred to as “2010–2014”), respectively [11]. The resulting database contained all BSI episodes and isolates obtained from a 10-year period between April 2007 and October 2017. Part of the data were presented in the 2 aforementioned survey reports [10, 11], as well as in reports of outbreak publications [10–15]. In addition to the methods described in Supplementary Box 1, detailed data of rainfall were retrieved for the entire study period, and additional molecular analysis of the DCS-associated factors and genetic typing of the isolates was performed (see “Molecular Analysis” below).

### Antibiotic Susceptibility Testing

Antibiotic susceptibility testing was carried out on the 2015–2017 isolates as described in Supplementary Document 1 with slight modifications. In brief, disk diffusion was performed with the exception of minimum inhibitory concentration (MIC) values testing for ciprofloxacin and azithromycin, for which the E-test macromethod (bioMérieux) was used [16]. Intermediate-susceptible isolates are grouped together with the resistant ones [17]. Where applicable, the databases of previous reports [10, 11] were reinterpreted according to the current guidelines of the Clinical and Laboratory Standards Institute (document M100-S28) [16]. Multidrug resistance (MDR) refers to the combined resistance to amoxicillin, chloramphenicol, and trimethoprim-sulfamethoxazole, and decreased ciprofloxacin susceptibility (DCS) refers to ciprofloxacin MIC values >0.06 mg/L and <1 mg/L. The term “full ciprofloxacin resistance” was reserved for MIC values ≥1 mg/mL [1, 16] and resistance to azithromycin was defined at MIC values >16 mg/L [16].

### Geographic Data and Data About Rainfall

In 2015, the 11 DRC provinces were split into the actual 26 provinces; for convenience of data display and comparison with previous publications, the former provinces were still used as the reference.

Satellite-based remote sensing techniques (Tropical Rainfall Measuring Mission [TRMM] data used for TRMM multisatellite precipitation analysis, Near Real-Time product version 7) were used to estimate the average accumulated monthly rainfall in millimeters per month [18]. Datasets can be accessed at https://disc-beta.gsfc.nasa.gov/. Calculations were performed using the R open source software (release 3.3.2) and linked to the actual provinces based on spatial data from the Royal Museum for Central Africa (Tervuren, Belgium). To obtain the monthly rainfall for the former provinces Equateur and Orientale, the average of the involved actual provinces was used.

### Molecular Analysis

DNA was extracted from the *Salmonella* Typhi isolates showing DCS and listed in Supplementary Table 2, using the Gentra PureGene Yeast/Bact Kit (Qiagen, Hilden, Germany) following the manufacturer’s guidelines, and whole genome sequencing was performed on an Illumina HiSeq platform (Illumina, San Diego, California). The sequencing data generated in this study (89 isolates) are available at the European Nucleotide Archive under the study accession number ERP109963. Four *Salmonella* Typhi had been sequenced in previous studies and the sequencing data are available under the sample accession numbers ERR349525, ERR352599, ERR357591 and PRJEB19771 [12, 19]. Molecular resistance mechanisms were determined from the raw Illumina sequencing data using ARIBA version 2.11.1 [20] with the Comprehensive Antibiotic Resistance Database version 2.0.2 [21]. Both known and novel variants of *gyr* and *par* genes conferring DCS were determined. Genome assemblies, constructed using de novo assembly [22], were used to determine the *Salmonella* Typhi Genotyphi genotype. Hereto, the Genotyphi implementation [23] or Pathogenwatch (https://pathogen.watch; Wellcome Trust Sanger Institute, Hinxton, Cambridge, United Kingdom) was used.

### Statistical Analysis

Unless otherwise stated, analysis and reporting were done for the first isolate per BSI episode and for those isolates that were available for reference testing at INRB/ITM. Statistical analysis was done with Stata software, version 12 (Stata Corp, College Station, Texas). Proportions were tested for significance using the χ^2^ test and differences in age distribution by the Wilcoxon-Mann-Whitney nonparametric test and the median test.

### Ethical Considerations

Ethical approval for the microbiological surveillance study was granted by the Institutional Review Board of ITM, the Ethics Committee of Antwerp University, and the Ministry of Health of the DRC.

## RESULTS

### Overview of Sampling

**Figure 1.**
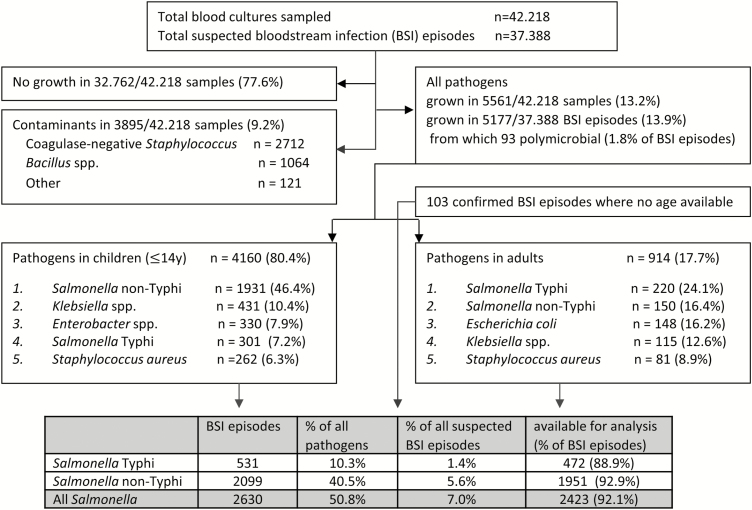
Breakdown of blood cultures sampled as part of the surveillance network, 2007–2017; unless otherwise stated, data are presented as first isolates per bloodstream infection episode (all blood cultures in a single patient within a 2-week period). In the top 5 of pathogens, the percentages represent the proportion of all pathogens in children or adults for whom information about age was available (n = 4160 and 914, respectively). Abbreviation: BSI, bloodstream infection.

**Figure 2.**
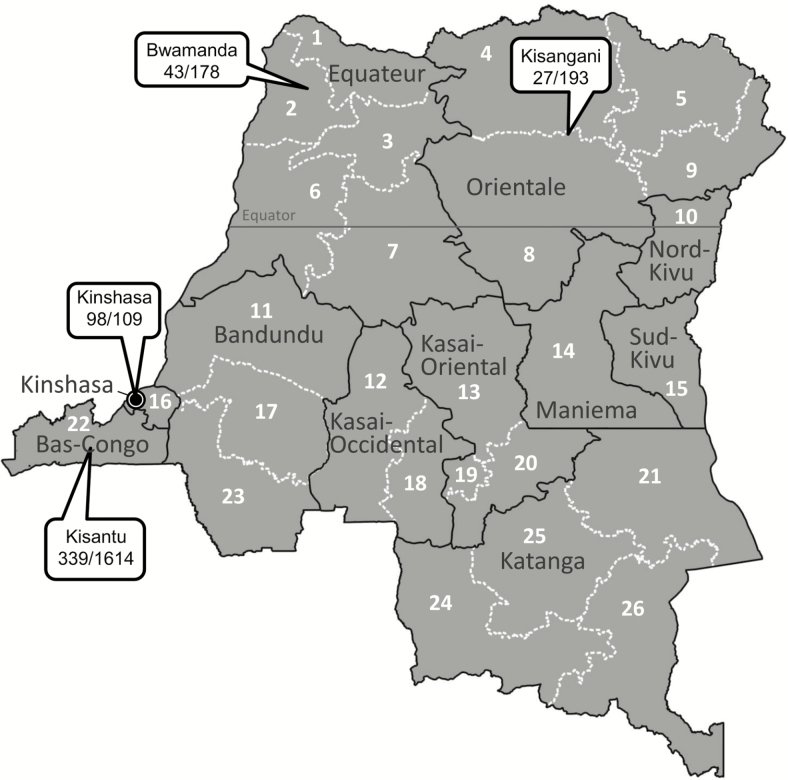
Map of the Democratic Republic of the Congo, with its (former) provinces, the main participating sampling sites in the microbiological surveillance network (call-outs), and the total numbers of *Salmonella* Typhi and *Salmonella* non-Typhi isolates. Additional numbers of isolates were 11 and 0, respectively, in Kasai-Occidental, 9 and 1 in Bandundu, 3 and 2 in Kasai Oriental, and 0 and 2 in Maniema. The new provinces are identified by numbers: 1, Nord-Ubangi; 2, Sud-Ubangi; 3, Mongala; 4, Bas-Uele; 5, Haut-Uele; 6, Equateur; 7, Tshuapa; 8, Tshopo; 9, Ituri; 10, Nord-Kivu; 11, Mai-Ndombe; 12, Kasai; 13, Sankuru; 14, Maniema; 15, Sud-Kivu; 16, Kinshasa; 17, Kwilu; 18, Kasai-Central; 19, Kasai-Oriental; 20, Lomami; 21, Tanganyika; 22, Kongo-Central; 23, Kwango; 24, Lualaba; 25, Haut-Lomami; 26, Haut-Katanga.

For the entire period (2007–2017), blood cultures sampled in 37 388 BSI episodes yielded 13.9% pathogens; for the period 2015–2017, the corresponding data were 13 657 and 12.0% (Figure 1). Yields of pathogens over time and sampling site were mostly between 10.0% and 15.0%. The highest yield (24.3%, Equateur 2011–2014) was associated with an outbreak of non-Typhi *Salmonella* infection [13]. The overall contamination rate was 9.2%; for the period 2015–2017, it was 7.17% with the lowest and highest rates at the sampling site Kisantu Hospital (Bas-Congo, 6.2%) and the surveillance network in Kinshasa (16.4%), respectively. Figure 2 shows the provinces of DRC with the main sampling sites and total numbers of *Salmonella* Typhi and non-Typhi isolates.

Overall, 4 of 5 suspected BSI episodes occurred in children (80.6% among 36.643 episodes for which data about age and year of sampling were available). The proportion of BSI episodes in children increased over time from 60.5% (5684/9398) in 2007–2010 to 90.2% (12 188/13 519) in 2015–2017, mainly by higher numbers of children sampled but—to a lesser extent—also due to lower numbers of adults sampled (1331 in 2015–2017 vs 2045 and 3714 in 2011–2014 and 2007–2010, respectively).

### Proportions of *Salmonella* Typhi Among Grown Blood Cultures

Overall, *Salmonella* Typhi accounted for 1.4% (531/37 388) and 10.3% (531/5177) of suspected and confirmed BSI, respectively (Figure 1). Among adults, *Salmonella* Typhi consistently ranked first among the pathogens recovered, representing 24.6% (110/447), 22.9% (67/293), and 24.3% (42/173) for the successive surveillance periods. Among the pathogens isolated from children, the proportion of *Salmonella* Typhi was lower—that is, 7.2% overall (Figure 1) and 11.4% (107/943), 7.1% (126/1774), and 4.7% (68/1442) for the successive surveillance periods. When subtracting neonates, the overall proportion of *Salmonella* Typhi among the pathogens increased to 7.8% (300/3831). *Salmonella* Typhi ranked third and second in frequency among pathogens isolated from children in Bas-Congo and Equateur, respectively. Non-Typhi *Salmonella* ranked first at all but 1 (Kinshasa) sampling site.

**Figure 3.**
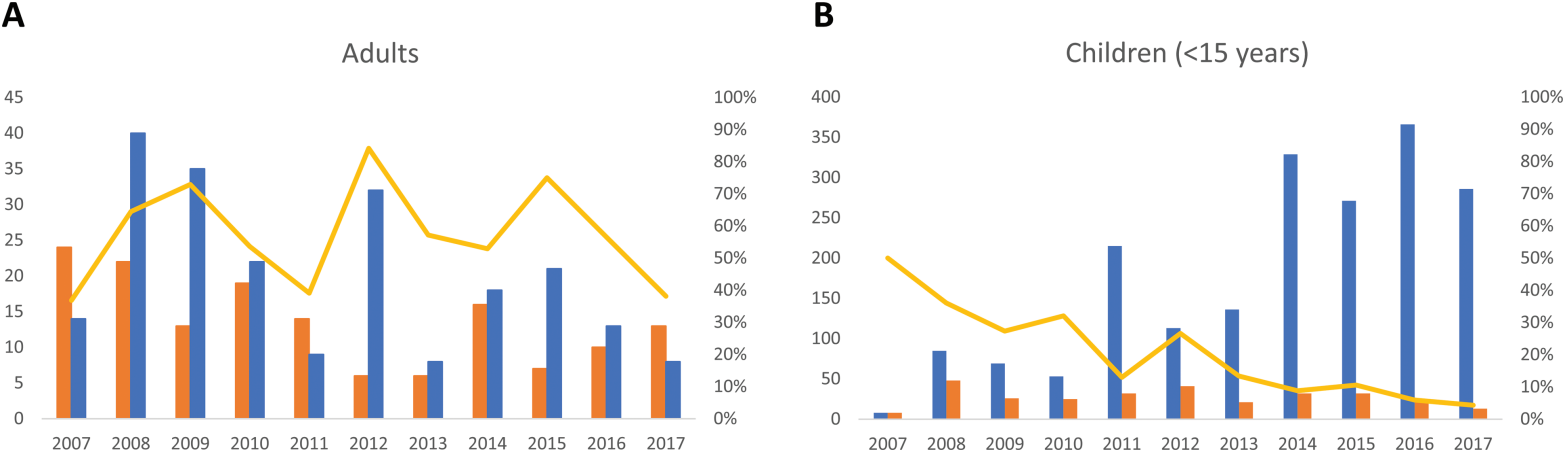
Over-time evolution of the frequency of *Salmonella* Typhi compared to non-Typhi *Salmonella* for adults (*A*) and children aged <15 years (*B*). The absolute numbers of *Salmonella* Typhi and non-Typhi *Salmonella* are shown by the blue and orange bars, respectively, and can be read on the left y-axis (note that the y-axis has a different scale for adults vs children). The yellow line represents the percentage of *Salmonella* Typhi vs all *Salmonella* isolates (Typhi and non-Typhi combined), reflected on the right y-axis.

Absolute numbers of *Salmonella* Typhi BSI were higher in children compared to adults. A total of 531 single isolates of *Salmonella* Typhi were obtained (301 [56.7%] from children vs 220 [41.4%] from adults; for 10 isolates no age was available [for details about age, see the next section]). Three recurrent BSI episodes were identified, occurring in children aged 12, 15, and 10 years old, respectively, with the recurrent episode occurring 3 weeks, 9 weeks, and 3 years after the initial BSI episode. A total of 472 (88.9%) of the isolates were available for further analysis (with missing isolates randomly distributed over time and sampling site). The numbers of *Salmonella* Typhi in adults showed little variation over time (Figure 3A), apart from small peaks in 2012 and 2015, which were randomly distributed over the sampling sites. The same observation was made for children; however, the rate of *Salmonella* Typhi vs non-Typhi *Salmonella* decreased sharply, in particular during the 2015–2017 surveillance period and mainly at the account of an increase of the non-Typhi *Salmonella* (Figure 3B).

### Age and Sex of Patients With *Salmonella* Typhi

**Figure 4.**
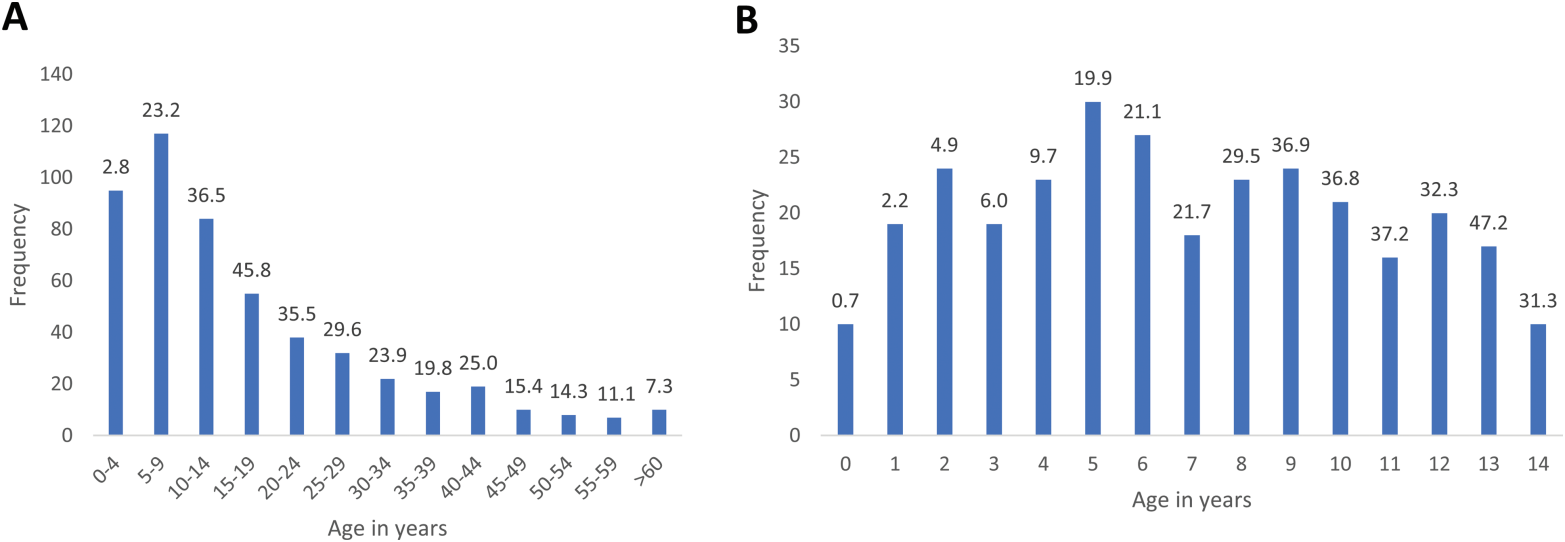
Distribution of *Salmonella* Typhi bloodstream infection episodes per age category (*A*) and per year of age for children (*B*), in absolute numbers (bars) and as a percentage of the total number of pathogens from patients with the same age (shown at top of bars). Data were generated for a total of 519 of 531 episodes for which data on age were recorded.

Figure 4 and Supplementary Figure 1 show the detailed age distribution. Absolute numbers of isolates in children were concentrated between 2 and 10 years of age and peaked around the age of 5. As a proportion of all isolates from BSI episodes, the highest rates were observed among children between 5 and 14 years old (19.9%–47.2%); 72.1% (217/301) and 31.6% (95/301) of *Salmonella* Typhi isolates were obtained in children <10 years and <5 years old, respectively, with 10 children <1 year old and the youngest 10 days old. The median age (interquartile range [IQR]) of patients with *Salmonella* Typhi BSI was 12 (IQR, 6–24) years, which was significantly higher than the corresponding data for the non-Typhi *Salmonella* (20 [IQR, 11–36] months) and all other BSI pathogens compiled (24 [IQR, 0–132] months) (*P* < .001). Median ages for *Salmonella* Typhi BSI were similar for the successive surveillance periods: 15 (IQR, 8–25), 10 (IQR, 5–21), and 10 (IQR, 5–21) years, respectively.

### Seasonality and Rainfall

**Figure 5.**
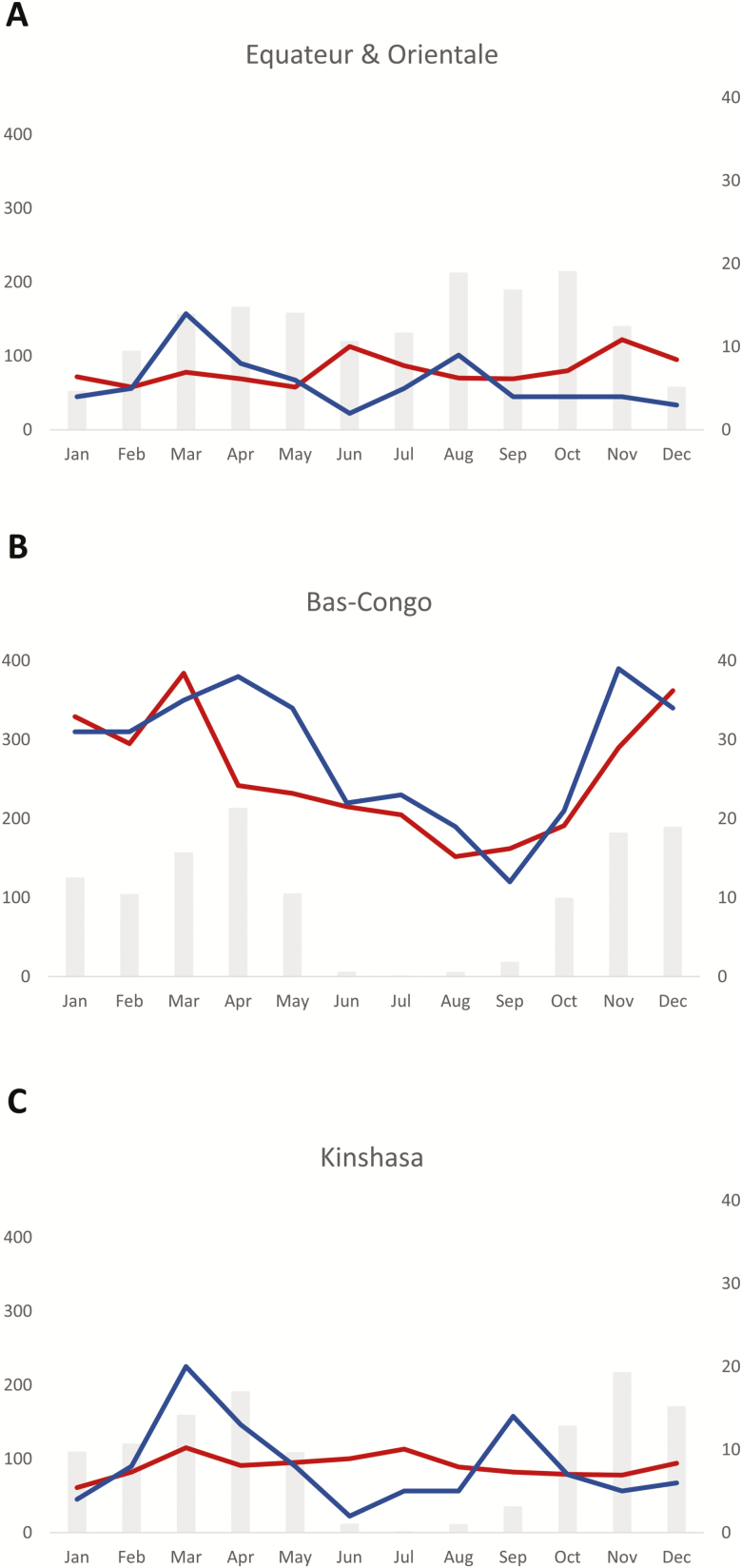
Relation between rainfall and frequency of *Salmonella* Typhi bloodstream infection in a rural area north (Orientale and Equateur, *A*) and south (Bas-Congo, *B*) of the equator, and an urban area (Kinshasa, *C*). The average rainfall for the 10-year surveillance period is displayed as gray bars and expressed in millimeters per month (left y-axis), the average numbers of *Salmonella* Typhi isolates (blue line), and bloodstream infection episodes caused by all pathogens (red line) per month, corresponding to the right y-axis.

The rainy season of Kinshasa and Bas-Congo (south of the equator) lasted from October through May and from March through November for Equateur and Orientale (north of the equator), respectively, although for the latter the difference between the rainy and dry season was less pronounced (Figure 5). In all provinces, *Salmonella* Typhi BSI was more frequent at the beginning and during the rainy season. In contrast to the nonfluctuating background of all-cause BSI in Kinshasa, Orientale, and Equateur, the all-cause BSI in Bas-Congo followed a seasonal pattern comparable to that of *Salmonella* Typhi.

### Antibiotic Resistance

As a percentage of *Salmonella* Typhi isolates for which antibiotic susceptibility was assessed, MDR was seen in 38.4% (n = 180), DCS in 24.5% (n = 115), and MDR combined with DCS in 11.9% (n = 56) of isolates, and azithromycin resistance was observed in 1 isolate (Table 1). One *Salmonella* Typhi isolate showing resistance to third-generation cephalosporins was found during an outbreak investigation in the province of Bandundu [12]. MDR rates over the successive surveillance periods were 39.9% (77/193), 36.9% (62/168), and 38.0% (41/108); corresponding DCS rates were 14.0% (27/193), 36.3% (61/168), and 25.0% (27/108), respectively. There was no apparent geographical concentration of MDR or DCS isolates. Overall, in almost half (204/422 [48.3%]) of the *Salmonella* Typhi BSI episodes, antibiotics had been given prior to admission (93/159 [58.5%], 71/153 [46.4%], and 40/110 [36.4%] for the successive surveillance periods). No statistically significant increased rate of MDR was observed when prehospital antibiotics were given (2015–2017: 38.5% MDR with and 37.7% MDR without prehospital antibiotic use; *P* = .936, χ^2^ test). There was a trend toward an increased rate of DCS if prehospital antibiotics had been given (2015–2017: 35.0% vs 18.6%; *P* = .054, χ^2^ test).

**Table 1.** Antibiotic Resistance Profile of *Salmonella* Typhi Isolates Recovered From Blood Cultures and for Which Antimicrobial Susceptibility Testing Was Done

Antibiotic	No. of Resistant Isolates	Percentage of Resistant Isolates
Ampicillin	329	70.0%
Trimethoprim-sulfamethoxazole	303	64.5%
Chloramphenicol	258	54.9%
Multidrug resistance	180	38.3%
DCS	115	24.5%
Azithromycin	2	0.4%
Third-generation cephalosporins	1	0.2%
Gentamicin	0	0%
Multidrug resistance + DCS	56	11.9%
DCS + azithromycin	1	0.2%
DCS + third-generation cephalosporins	1	0.2%

The total number of isolates available for reference testing was 470.

Abbreviation: DCS, decreased ciprofloxacin susceptibility.

### Decreased Ciprofloxacin Susceptibility and Genotyphi Genotype

**Figure 6.**
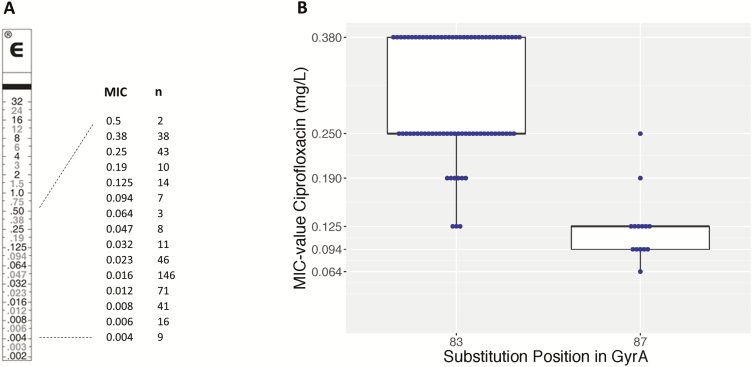
*A*, Minimum inhibitory concentration (MIC) values of ciprofloxacin for 465 isolates of *Salmonella* Typhi with decreased ciprofloxacin susceptibility, along an E-test macromethod strip. *B*, Box plot with ciprofloxacin MIC values for S83F and D87G amino acid substitutions in gyrA. Abbreviation: MIC, minimum inhibitory concentration.

Figure 6A shows the ciprofloxacin MIC value distribution of the *Salmonella* Typhi isolates from the entire surveillance period. No full resistance was noted. A nonsynonymous mutation in the *gyrA* or *gyrB* genes was identified for all 93 confirmed DCS *Salmonella* Typhi isolates (Supplementary Table 2), with 1 isolate showing 2 nonsynonymous mutations in *gyrA*. A substitution of gyrA S83 was seen in 91.7% of isolates (n = 76, of which 72 were S83F and 4 were S83Y); another 15.1% had a substitution of D87 in gyrA (n = 14, of which 13 were D87G and 1 was D87Y) and 1.1% showed a novel gyrA A119E substitution. A substitution of gyrB E466D and S464Y was each observed in 1.1% (n = 1). Strikingly, the gyrA S83F substitution resulted in higher MIC values (clustering around 0.25 and 0.38 mg/L compared with the substitution at gyrA D87G, which clustered at 0.094 and 0.125 mg/L; Figure 6B). The majority (97.8 %) of the sequenced DCS isolates belonged to Genotyphi genotype 2.5.1, whereas 1.1% (n = 1) had genotype 4.3.1 and 1.1% (n = 1) had genotype 2.1, also known as lineage H58.

## DISCUSSION

This study gives an overview of *Salmonella* Typhi BSI in the light of a decade of microbiological surveillance in DRC, a country where diagnostic microbiological facilities are almost nonexistent [24]. The embedment in the routine patient management in a low-resource setting faced the well-known technical, logistical, and human resource problems [25, 26]. Furthermore, accessibility, local needs of patient care, and security concerns directed the selection of the healthcare facilities. Besides representativeness and coverage, the healthcare facility–based approach had other limitations. Most important, it was not possible to calculate and monitor population-based incidence in the absence of solid demographic data (census, health utilization data) [4, 6]. Second, sampling and laboratory workup were done by local staff and may have accounted for higher rates of contaminants and more missing data compared to a research setting. Finally, the health facility–based approach was dependent on the health-seeking behavior and the referral itinerary of patients with a consequently low spatial resolution (presently limited to the health center or village level).

As to its strengths, the healthcare facility surveillance approach was free of charge for patients and integrated in clinical care. This allowed us to reach many patients in a clinical context and was a most cost-effective strategy to obtain a consistently large number of isolates—among them also those of other bacterial species. The INRB team managed to maintain the network over a long time period, with a high proportion of isolates available for reference testing. Despite a too high contamination rate (9%), the pathogen rate remained high and within the expected ranges over time and site.

*Salmonella* Typhi consistently ranked first as the cause of BSI in adults, indicative of its frequent occurrence of typhoid fever in the population, as laboratory-confirmed *Salmonella Typhi* in hospital admitted patients represent only a small fraction of all cases [27]. Despite the overrepresentation of children sampled, *Salmonella* Typhi accounted for 7.2% of BSIs in children, most of them in the first decade of life, comparable to recent observations in East and West Africa [5, 28]. The predominance of *Salmonella* Typhi in young children highlights the importance of identifying the most effective vaccination strategy. Since March 2018, the WHO has recommended the new typhoid conjugate vaccine for single-dose immunization of children from 6 months of age onward [29]. Although typhoid fever occurred perennially, there was a seasonal trend with a moderate increase in the rainy season as previously described in other African countries [30, 31]. The contamination of surface and drinking water is the most plausible explanation, although the component of malnutrition, which in DRC occurs mainly during the rainy season [32], might play a role.

MDR and DCS were found in approximately 1 of 3 and 4 isolates, respectively; 1 in 8 isolates showed combined DCS and MDR. After an increase of DCS in 2011–2014 compared to 2007–2010, the rate of DCS slightly decreased (24.6%). The rate of MDR remained stable in 2015–2017 (37.9%). The observed MIC values for gyrA substitutions are comparable to those previously published from Asia [33, 34]. The frequency of antimicrobial resistance was high and widespread, with no geographical clustering on province level. Of note, the *Salmonella* Typhi with MDR and cephalosporin resistance previously reported from the DRC belonged to the same clade (genotype 2.5.1) as the majority of the presently assessed DCS isolates, which highlights the relevance of ongoing surveillance and the implementation of vaccination [12].

In conclusion, the established microbiological surveillance network and the high numbers of *Salmonella* Typhi BSI episodes in children elicit the DRC as a candidate to study the efficacy of the introduction of the typhoid conjugate vaccine [4]. MDR and DCS remained a major threat over the whole decade and warrant sustainable surveillance efforts [35].

## Supplementary Data

Supplementary materials are available at *Clinical Infectious Diseases* online. Consisting of data provided by the authors to benefit the reader, the posted materials are not copyedited and are the sole responsibility of the authors, so questions or comments should be addressed to the corresponding author.

ciy1116_suppl_Supplementary_Figure_1

ciy1116_suppl_Supplementary_Table_1

ciy1116_suppl_Supplementary_Table_2

ciy1116_suppl_Supplementary_Document_1

ciy1116_suppl_Supplementary_Figure_Legend

## Notes

***Acknowledgments.*** The authors thank all local clinical and laboratory staff for their contribution and dedication to the microbiological surveillance network. The authors acknowledge the National Aeronautics and Space Administration’s Goddard Earth Sciences Data and Information Services Center for providing full access to the precipitation datasets exploited in this study.

***Financial support.*** This work was funded by the Belgian Directorate of Development Cooperation (DGD) through Project 2.01 of the Third Framework Agreement between the Belgian DGD and the Institute of Tropical Medicine, Belgium, and by the Baillet-Latour fund. E. M. has a scholarship from the Fonds de la Recherche Scientifique, Belgium.

***Supplement sponsorship.*** This supplement is sponsored by the Center for Vaccine Development and Global Health (CVD) at the University of Maryland School of Medicine.

***Potential conflicts of interest.*** All authors: No reported conflicts. All authors have submitted the ICMJE Form for Disclosure of Potential Conflicts of Interest. Conflicts that the editors consider relevant to the content of the manuscript have been disclosed.

## References

[1] Crump JA , Sjölund-Karlsson M, Gordon MA, Parry CM. Epidemiology, clinical presentation, laboratory diagnosis, antimicrobial resistance, and antimicrobial management of invasive *Salmonella* infections. Clin Microbiol Rev 2015; 28:901–37. 26180063 10.1128/CMR.00002-15 PMC4503790

[2] Buckle GC , Walker CLF, Black RE. Typhoid fever and paratyphoid fever: systematic review to estimate global morbidity and mortality for 2010. J Glob Health 2012; 2:10401. 10.7189/jogh.02.010401 PMC3484760 23198130

[3] Tacconelli E , Carrara E, Savoldi A, et al Discovery, research, and development of new antibiotics: the WHO priority list of antibiotic-resistant bacteria and tuberculosis. Lancet Infect Dis 2017; 3099:1–10. 10.1016/S1473-3099(17)30753-3 29276051

[4] Marks F , von Kalckreuth V, Aaby P, et al Incidence of invasive *Salmonella* disease in sub-Saharan Africa: a multicentre population-based surveillance study. Lancet Glob Health 2017; 5:e310–23. 28193398 10.1016/S2214-109X(17)30022-0 PMC5316558

[5] Jeon HJ , Pak GD, Im J, et al Determining the best immunization strategy for protecting African children against invasive *Salmonella* disease. Clin Infect Dis 2018; 67:1824–30. 29746615 10.1093/cid/ciy386 PMC6260167

[6] Guiraud I , Post A, Diallo SN, et al Population-based incidence, seasonality and serotype distribution of invasive salmonellosis among children in Nanoro, rural Burkina Faso. PLoS One 2017; 12:1–17. 10.1371/journal.pone.0178577 PMC5503169 28692655

[7] Antillón M , Warren JL, Crawford FW, et al The burden of typhoid fever in low- and middle-income countries: a meta-regression approach. PLoS Negl Trop Dis 2017; 11:1–21. 10.1371/journal.pntd.0005376 PMC5344533 28241011

[8] Muyembe-Tamfum JJ , Veyi J, Kaswa M, Lunguya O, Verhaegen J, Boelaert M. An outbreak of peritonitis caused by multidrug-resistant *Salmonella* Typhi in Kinshasa, Democratic Republic of Congo. Travel Med Infect Dis 2009; 7:40–43. 19174300 10.1016/j.tmaid.2008.12.006

[9] Ali E , Van Den Bergh R, D’hondt R, et al Localised transmission hotspots of a typhoid fever outbreak in the Democratic Republic of Congo. Pan Afr Med J 2017; 28:1–9. 10.11604/pamj.2017.28.179.10208 PMC5847255 29541325

[10] Lunguya O , Lejon V, Phoba MF, et al *Salmonella* Typhi in the Democratic Republic of the Congo: fluoroquinolone decreased susceptibility on the rise. PLoS Negl Trop Dis 2012; 6:3–8. 10.1371/journal.pntd.0001921 PMC3499407 23166855

[11] Kalonji LM , Post A, Phoba MF, et al Invasive *Salmonella* infections at multiple surveillance sites in the Democratic Republic of the Congo, 2011–2014. Clin Infect Dis 2015; 61:S346–53. 26449951 10.1093/cid/civ713

[12] Phoba M-F , Barbé B, Lunguya O, et al *Salmonella enterica* serovar Typhi producing CTX-M-15 extended spectrum β-lactamase in the Democratic Republic of the Congo. Clin Infect Dis 2017; 65:1229–31. 29136410 10.1093/cid/cix342 PMC5849100

[13] Phoba MF , De Boeck H, Ifeka BB, et al Epidemic increase in *Salmonella* bloodstream infection in children, Bwamanda, the Democratic Republic of Congo. Eur J Clin Microbiol Infect Dis 2014; 33:79–87. 23975545 10.1007/s10096-013-1931-8

[14] Phoba M-F , Lunguya O, Mayimon DV, et al Multidrug-resistant *Salmonella enterica*, Democratic Republic of the Congo. Emerg Infect Dis 2012; 18:1692–4. 23017665 10.3201/eid1810.120525 PMC3471636

[15] Falay D , Kuijpers LMF, Phoba MF, et al Microbiological, clinical and molecular findings of non-typhoidal *Salmonella* bloodstream infections associated with malaria, Oriental Province, Democratic Republic of the Congo. BMC Infect Dis 2016; 16:271. 27286886 10.1186/s12879-016-1604-1 PMC4902913

[16] Clinical and Laboratory Standards Institute. Performance standards for antimicrobial susceptibility testing. Wayne, PA: CLSI, 2018.

[17] Clinical and Laboratory Standards Institute. M39-A4 analysis and presentation of cumulative antimicrobial susceptibility test data; approved guideline, 4th ed. Wayne, PA: CLSI, 2014.

[18] Huffman GJ , Bolvin DT, Nelkin EJ, et al The TRMM multisatellite precipitation analysis (TMPA): quasi-global, multiyear, combined-sensor precipitation estimates at fine scales. J Hydrometeorol 2007; 8:38–55.

[19] Wong VK , Baker S, Pickard DJ, et al Phylogeographical analysis of the dominant multidrug-resistant H58 clade of *Salmonella* Typhi identifies inter- and intracontinental transmission events. Nat Genet 2015; 47:632–9. 25961941 10.1038/ng.3281 PMC4921243

[20] Hunt M , Mather AE, Sánchez-Busó L, et al ARIBA: rapid antimicrobial resistance genotyping directly from sequencing reads. Microb Genomics 2017; 3:e000131. 10.1099/mgen.0.000131 PMC5695208 29177089

[21] McArthur AG , Waglechner N, Nizam F, et al The comprehensive antibiotic resistance database. Antimicrob Agents Chemother 2013; 57:3348–57. 23650175 10.1128/AAC.00419-13 PMC3697360

[22] Page AJ , De Silva N, Hunt M, et al Robust high-throughput prokaryote de novo assembly and improvement pipeline for Illumina data. Microb Genom 2016; 2:e000083. 28348874 10.1099/mgen.0.000083 PMC5320598

[23] Wong VK , Baker S, Connor TR, et al An extended genotyping framework for *Salmonella enterica* serovar Typhi, the cause of human typhoid. Nat Commun 2016; 7:12827. 27703135 10.1038/ncomms12827 PMC5059462

[24] Lunguya O , Phoba MF, Mundeke SA, et al The diagnosis of typhoid fever in the Democratic Republic of the Congo. Trans R Soc Trop Med Hyg 2012; 106:348–55. 22551639 10.1016/j.trstmh.2012.03.006

[25] Barbé B , Yansouni CP, Affolabi D, Jacobs J. Implementation of quality management for clinical bacteriology in low-resource settings. Clin Microbiol Infect 2017; 23:426–33. 28506781 10.1016/j.cmi.2017.05.007

[26] Ombelet S , Ronat JB, Walsh T, et al Clinical bacteriology in low-resource settings: today’s solutions. Lancet Infect Dis 2018; 3099:1–11. 10.1016/S1473-3099(18)30093-8 29519767

[27] Crump JA , Youssef FG, Luby SP, et al Estimating the incidence of typhoid fever and other febrile illnesses in developing countries. Emerg Infect Dis 2003; 9:539–44. 12737736 10.3201/eid0905.020428 PMC2972755

[28] Mogasale V , Maskery B, Ochiai RL, et al Burden of typhoid fever in low-income and middle-income countries: a systematic, literature-based update with risk-factor adjustment. Lancet Glob Health 2014; 2:e570–80. 25304633 10.1016/S2214-109X(14)70301-8

[29] World Health Organization. Typhoid vaccines: WHO position paper, March 2018—recommendations. Vaccine 2018; 153–72. 10.1016/j.vaccine.2018.04.022 29661581

[30] Labi A-K , Obeng-Nkrumah N, Addison NO, Donkor ES, Donkor ES. *Salmonella* blood stream infections in a tertiary care setting in Ghana. BMC Infect Dis 2014; 14:3857. 25528352 10.1186/s12879-014-0697-7 PMC4297363

[31] Breiman RF , Cosmas L, Njuguna H, et al Population-based incidence of typhoid fever in an urban informal settlement and a rural area in Kenya: implications for typhoid vaccine use in Africa. PLoS One 2012; 7. 10.1371/journal.pone.0029119 PMC3261857 22276105

[32] Kismul H , Schwinger C, Chhagan M, Mapatano M, Van den Broeck J. Incidence and course of child malnutrition according to clinical or anthropometrical assessment: a longitudinal study from rural DR Congo. BMC Pediatr 2014; 14:22. 24467733 10.1186/1471-2431-14-22 PMC3915030

[33] Chau TT , Campbell JI, Galindo CM, et al Antimicrobial drug resistance of *Salmonella enterica* serovar Typhi in Asia and molecular mechanism of reduced susceptibility to the fluoroquinolones. Antimicrob Agents Chemother 2007; 51:4315–23. 17908946 10.1128/AAC.00294-07 PMC2167998

[34] Parry CM , Thuy CT, Dongol S, et al Suitable disk antimicrobial susceptibility breakpoints defining *Salmonella enterica* serovar Typhi isolates with reduced susceptibility to fluoroquinolones. Antimicrob Agents Chemother 2010; 54:5201–8. 20837759 10.1128/AAC.00963-10 PMC2981260

[35] Parry CM , Threlfall EJ. Antimicrobial resistance in typhoidal and nontyphoidal salmonellae. Curr Opin Infect Dis 2008; 21:531–8. 18725804 10.1097/QCO.0b013e32830f453a

