# Correction to: *Salmonella Typhi* From Blood Cultures in the Democratic Republic of the Congo: A 10-Year Surveillance

**DOI:** 10.1093/cid/ciac145

**Published:** 2022-03-24

**Authors:** Bieke Tack, Marie-France Phoba, Sandra Van Puyvelde, Lisette M Kalonji, Liselotte Hardy, Barbara Barbé, Marianne A B Van der Sande, Elise Monsieurs, Stijn Deborggraeve, Octavie Lunguya, Jan Jacobs

**Affiliations:** 1 Department of Clinical Sciences, Institute of Tropical Medicine, Antwerp; 2 Department of Microbiology and Immunology, KU Leuven, Belgium; 3 Department of Microbiology, National Institute for Biomedical Research; 4 Department of Microbiology, University Teaching Hospital, Kinshasa, Democratic Republic of the Congo, Antwerp, Belgium; 5 Department of Biomedical Sciences, Institute of Tropical Medicine, Antwerp, Belgium; 6 Wellcome Trust Sanger Institute, Hinxton, United Kingdom; 7 Department of Public Health, Institute of Tropical Medicine, Antwerp, Belgium; 8 Julius Center for Health Sciences and Primary Care, Global Health Centre, Utrecht University, The Netherlands; 9 Royal Museum for Central Africa, Tervuren; 10 Department of Geography, University of Liège, Belgium

In the original publication of this manuscript [Tack B, Phoba M, Puyvelde SV et al. *Salmonella Typhi* From Blood Cultures in the Democratic Republic of the Congo: A 10-Year Surveillance. Clin Infect Dis. Volume 68 Supplement 2; March 15 2019, S130-S137. https://doi.org/10.1093/cid/ciy1116], the article’s Supplementary Table 2, the study’s list of sequenced isolates and their metadata, was missing. It is hosted with this notice for review, and the authors regret this error.

**Table:** 

**Sample Name**	**Serotyping UTLM**	**MLST ST**	**GENOTYPHI GENOTYPE**	**gyrA_8.D87G**	**gyrA_8.S83F**	**gyrB_1.E466D**	**gyrA_8.A119E**	**gyrA_8.D538N**	**gyrA_8.D87Y**	**gyrA_8.S83Y**	**gyrB_1.I471S**	**gyrB_1.S464Y**	**NrSandra**	**NrorigdbID**	**Provenance2906**	**Provincedboriginal**	**AgeA**	**Année**	**CIPE1908**
3525_3	Typhi	2	2.5.1	no	yes	no	no	no	no	no	no	no	3525_3	6134	KISANTU	BAS-CONGO	7	2011	.25
3592_3	Typhi	2	2.5.1	no	yes	no	no	no	no	no	no	no	3592_3	6201	KISANTU	BAS-CONGO	23	2011	.25
3632_3	Typhi	2	2.5.1	no	yes	no	no	no	no	no	no	no	3632_3	6241	KISANTU	BAS-CONGO	11	2011	.38
3524_3	Typhi	2	2.5.1	no	yes	no	no	no	no	no	no	no	3524_3	6133	KISANTU	BAS-CONGO	36	2011	.38
3808_3	Typhi	2	2.5.1	no	yes	no	no	no	no	no	no	no	3808_3	7556	KISANTU	BAS-CONGO	20	2011	.38
3841_3	Typhi	2	2.5.1	no	yes	no	no	no	no	no	no	no	3841_3	7589	KISANTU	BAS-CONGO	42	2011	.19
3872_3	Typhi	2	2.5.1	no	no	no	yes	no	no	no	no	no	3872_3	7620	KISANTU	BAS-CONGO	4	2011	.38
3874_3	Typhi	2	2.5.1	no	yes	no	no	no	no	no	no	no	3874_3	0	KISANTU	BAS-CONGO	33	2011	.38
4209_3	Typhi	2	2.5.1	no	yes	no	no	no	no	no	no	no	4209_3	7957	KISANTU	BAS-CONGO	13	2011	.38
4343_3	Typhi	2	2.5.1	no	yes	no	no	no	no	no	no	no	4343_3	9079	KISANTU	BAS-CONGO	6	2011	.38
4421_3	Typhi	2	2.5.1	no	yes	no	no	no	no	no	no	no	4421_3	9157	KISANTU	BAS-CONGO	21	2012	.38
4441_3	Typhi	2	2.5.1	no	yes	no	no	no	no	no	no	no	4441_3	9177	KISANTU	BAS-CONGO	45	2012	.38
22246_3	Typhi	2	2.5.1	no	yes	no	no	no	no	no	no	no	22246_3	2483	HSLK	KONGO CENTRAL	29	2017	.25
4586_3	Typhi	2	2.5.1	no	yes	no	no	no	no	no	no	no	4586_3	9322	KISANTU	BAS-CONGO	30	2012	.38
5273_3	Typhi	2	2.5.1	no	yes	no	no	no	no	no	no	no	5273_3	10567	KISANTU	BAS-CONGO	42	2012	.25
5741	Typhi	2	2.5.1	no	yes	no	no	no	no	no	no	no	5741_3	11035	KISANTU	BAS-CONGO	3	2012	.38
5893_3	Typhi	2	2.5.1	no	yes	no	no	no	no	no	no	no	5893_3	11187	KISANTU	BAS-CONGO	8	2012	.38
5885_3	Typhi	2	2.5.1	no	yes	no	no	no	no	no	no	no	5885_3	11179	KISANTU	BAS-CONGO	6	2012	.38
5918_3	Typhi	2	2.5.1	no	yes	no	no	no	no	no	no	no	5918_3	11212	KISANTU	BAS-CONGO	19	2012	.38
5927_3	Typhi	2	2.5.1	no	yes	no	no	no	no	no	no	no	5927_3	11221	KISANTU	BAS-CONGO	19	2012	.38
5867_3	Typhi	2	2.5.1	no	yes	no	no	no	no	no	no	no	5867_3	11161	KISANTU	BAS-CONGO	10	2012	.38
2697_13	Typhi	2	2.1	no	no	no	no	no	yes	no	no	no	2697_13	12803	VDP	ORIENTALE	4	2013	0.19
22271_3	Typhi	2	2.5.1	no	yes	no	no	no	no	no	no	no	22271_3	2508	HSLK	KONGO CENTRAL	24M	2017	.25
6591_3	Typhi	2	2.5.1	no	no	yes	no	no	no	no	no	no	6591_3	13143	KISANTU	BAS-CONGO	5	2013	.064
6637_3	Typhi	2	2.5.1	no	yes	no	no	no	no	no	no	no	6637_3	13189	KISANTU	BAS-CONGO	57	2013	.38
6657_3	Enteritidis	2	2.5.1	no	yes	no	no	no	no	no	no	no	6657_3	13209	KISANTU	BAS-CONGO	7	2013	.38
6976_3	Typhi	2	2.5.1	no	yes	no	no	no	no	no	no	no	6976_3	13627	KISANTU	BAS-CONGO	42	2013	.38
7196_3	Typhi	2	2.5.1	no	yes	no	no	no	no	no	no	no	7196_3	13847	KISANTU	BAS-CONGO	6	2013	.38
7217_3	Typhi	2	2.5.1	no	yes	no	no	no	no	no	no	no	7217_3	13868	KISANTU	BAS-CONGO	3	2013	.38
22272_3	Typhi	2	2.5.1	no	yes	no	no	no	no	no	no	no	22272_3	2509	HSLK	KONGO CENTRAL	7	2017	0.38
22320_3	Typhi	2	2.5.1	no	yes	no	no	no	no	no	no	no	22320_3	2557	HSLK	KONGO CENTRAL	35	2017	.25
22323_3	Typhi	2	2.5.1	no	yes	no	no	no	no	no	no	no	22323_3	2560	HSLK	KONGO CENTRAL	ND	2017	.25
10613_17	Typhi	2	2.5.1	yes	no	no	no	no	no	no	no	no	10613_17	3842	CUK	KINSHASA	27	2017	.094
22545_3	Enteritidis	2	2.5.1	no	yes	no	no	no	no	no	no	no	22545_3	2782	HSLK	KONGO CENTRAL	46M	2017	.38
22493_3	Enteritidis	2	2.5.1	no	yes	no	no	no	no	no	no	no	22493_3	2730	HSLK	KONGO CENTRAL	31	2017	.25
23164_3	Typhi	2	2.5.1	no	yes	no	no	no	no	no	no	no	23164_3	3401	HSLK	KONGO CENTRAL	32	2017	.25
6807_3	Typhi	2	2.5.1	no	no	no	no	no	no	no	no	yes	6807_3	13458	KISANTU	BAS-CONGO	6	2013	.19
7569_3	Typhi	2	2.5.1	no	yes	no	no	no	no	no	no	no	7569_3	14220	KISANTU	BAS-CONGO	29	2013	.38
7818_3	Typhi	2	2.5.1	no	yes	no	no	no	no	no	no	no	7818_3	14740	KISANTU	BAS-CONGO	6	2013	.38
9202_14	Typhi	2	2.5.1	no	yes	no	no	no	no	no	no	no	9202_14	366	MBANZA VELELA/ZS KINGASANI	KINSHASA	16	2014	.25
9207_14	Typhi	2	2.5.1	no	yes	no	no	no	no	no	no	no	9207_14	371	CS LONDOLOBE/ZS KINGASANI	KINSHASA	14	2014	.25
9210_14	Typhi	2	2.5.1	no	yes	no	no	no	no	no	no	no	9210_14	374	CS LONDOLOBE/ZS KINGASANI	KINSHASA	10	2014	.25
9213_14	Typhi	2	2.5.1	no	yes	no	no	no	no	no	no	no	9213_14	377	DOMICILE/ZS KINGASANI	KINSHASA	11	2014	.25
9214_14	Typhi	2	2.5.1	no	yes	no	no	no	no	no	no	no	9214_14	378	CS KIMBANGUISTE/ZS KINGASANI	KINSHASA	32	2014	.25
9238_14	Typhi	2	2.5.1	no	yes	no	no	no	no	no	no	no	9238_14	402	CS LONDOLOBE/ZS KINGASANI	KINSHASA	44	2014	.25
9262_14	Typhi	2	2.5.1	no	yes	no	no	no	no	no	no	no	9262_14	426	CS LONDOLOBE/ZS KINGASANI	KINSHASA	23	2014	.25
9267_14	Typhi	2	2.5.1	no	yes	no	no	no	no	no	no	no	9267_14	431	CS LONDOLOBE/ZS KINGASANI	KINSHASA	12	2014	.25
9305_14	Typhi	2	2.5.1	no	yes	no	no	no	no	no	no	no	9305_14	469	HGR MOSANGO/ZS KINGASANI	KINSHASA	ND	2014	.38
8357_3	Typhi	2	2.5.1	yes	no	no	no	no	no	no	no	no	8357_3	497	HSLK	BAS-CONGO	29M	2014	.094
21602_3	Typhi	2	2.5.1	no	yes	no	no	no	no	no	no	no	21602_3	1707	HSLK	KONGO CENTRAL	8	2017	.38
8375_3	Typhi	2	2.5.1	yes	no	no	no	no	no	no	no	no	8375_3	515	HSLK	BAS-CONGO	40	2014	.125
8675_3	Typhi	2	2.5.1	yes	no	no	no	no	no	no	no	no	8675_3	815	HSLK	BAS-CONGO	27	2014	0.12
9099_3	Typhi	2	2.5.1	yes	no	no	no	no	no	no	no	no	9099_3	1238	HSLK	BAS-CONGO	7	2014	.12
8852_3	Typhi	2	2.5.1	yes	no	no	no	no	no	no	no	no	8852_3	992	HSLK	BAS-CONGO	23M	2014	.06
9311_3	Typhi	2	2.5.1	yes	no	no	no	no	no	no	no	no	9311_3	1450	HSLK	BAS-CONGO	2	2014	.25
9392_3	Typhi	2	2.5.1	no	yes	no	no	no	no	no	no	no	9392_3	1531	HSLK	BAS-CONGO	10J	2014	.25
9728_3	Typhi	2	2.5.1	no	yes	no	no	no	no	no	no	no	9728_3	2579	HSLK	BAS-CONGO	30	2014	.25
10474_3	Typhi	2	2.5.1	no	yes	no	no	no	no	no	no	no	10474_3	4337	HSLK	BAS-CONGO	27M	2014	.12
11280_3	Typhi	2	2.5.1	no	yes	no	no	no	no	no	no	no	11280_3	5143	HSLK	BAS-CONGO	ND	2014	.25
12942_3	Typhi	2	2.5.1	no	yes	no	no	no	no	no	no	no	12942_3	6919	HSLK	BAS-CONGO	10	2015	.25
9642_15	Typhi	2	2.5.1	no	no	no	no	no	no	yes	no	no	9642_15	7739	HGR BWAMANDA	EQUATEUR	28	2015	.125
9921_15	Typhi	2	2.5.1	no	no	no	no	no	no	yes	no	no	9921_15	8887	HGR BWAMANDA	EQUATEUR	9	2015	.19
14708_3	Typhi	2	2.5.1	yes	no	no	no	no	no	no	no	no	14708_3	9201	HSLK	BAS-CONGO	30	2015	.094
14824_3	Typhi	2	2.5.1	yes	no	no	no	no	no	no	no	no	14824_3	9317	HSLK	BAS-CONGO	29	2015	.094
14381_3	Typhi	2	2.5.1	no	yes	no	no	no	no	no	no	no	14381_3	8627	HSLK	BAS-CONGO	15	2015	.19
13231_3	Typhi	2	2.5.1	no	no	no	no	no	no	yes	no	no	13231_3	7208	HSLK	BAS-CONGO	24	2015	.25
15601_3	Typhi	2	2.5.1	yes	no	no	no	no	no	no	no	no	15601_3	10302	HSLK	BAS-CONGO	5	2015	.094
15824_3	Typhi	2	2.5.1	no	yes	no	no	no	no	no	no	no	15824_3	10525	HSLK	BAS-CONGO	28	2016	.125
16428_3	Typhi	2	2.5.1	yes	no	no	no	no	no	no	no	no	16428_3	11435	HSLK	BAS-CONGO	22	2016	.125
16820_3	Typhi	2	2.5.1	no	yes	no	no	no	no	no	no	no	16820_3	11827	HSLK	BAS-CONGO	28	2016	.25
17421_3	Typhi	2	2.5.1	yes	no	no	no	no	no	no	no	no	17421_3	12444	HSLK	KONGO CENTRAL	41	2016	.125
10456_16	Typhi	1	4.3.1	no	no	no	no	yes	no	yes	no	no	10456_16	14150	CUK	EQUATEUR	22	2016	.25
5706_4	Typhi	2	2.5.1	no	yes	no	no	no	no	no	no	no	5706_4	58	HGRK	TSHOPO	5	2017	.25
18858_3	Typhi	2	2.5.1	no	yes	no	no	no	no	no	no	no	18858_3	14492	HSLK	KONGO CENTRAL	45	2016	.25
20298_3	Typhi	2	2.5.1	no	yes	no	no	no	no	no	no	no	20298_3	403	HSLK	KONGO CENTRAL	7	2017	.25
20304_3	Typhi	2	2.5.1	yes	no	no	no	no	no	no	no	no	20304_3	409	HSLK	KONGO CENTRAL	6	2017	.125
20867_3	Typhi	2	2.5.1	no	yes	no	no	no	no	no	no	no	20867_3	972	HSLK	KONGO CENTRAL	5	2017	.38
22066_3	Typhi	2	2.5.1	no	yes	no	no	no	no	no	no	no	22066_3	2303	HSLK	KONGO CENTRAL	10	2017	.25
2228_09	Typhi	2	2.5.1	no	yes	no	no	no	no	no	no	no	2228_09	547	CS MOBENGI	KINSHASA	9	2009	0.19
3673	Typhi	2	2.5.1	no	yes	no	no	no	no	no	no	no	3673	661	CUK	KINSHASA	32	2009	0.25
2267_3	Typhi	2	2.5.1	no	yes	no	no	no	no	no	no	no	2267_3	1625	KISANTU	BAS-CONGO	15	2009	0.38
2366	Typhi	2	2.5.1	no	yes	no	no	no	no	no	no	no	2366_3	0	KISANTU	BAS-CONGO	40	2009	0.25
2539_3	Typhi	2	2.5.1	no	yes	no	no	no	no	no	no	no	2539_3	3212	KISANTU	BAS-CONGO	30	2010	0.38
2628_3	Typhi	2	2.5.1	no	yes	no	no	no	no	no	no	no	2628_3	3301	KISANTU	BAS-CONGO	17	2010	0.38
2654_3	Typhi	2	2.5.1	no	yes	no	no	no	no	no	no	no	2654_3	3327	KISANTU	BAS-CONGO	5	2010	0.19
2656_3	Typhi	2	2.5.1	no	yes	no	no	no	no	no	no	no	2656_3	3329	KISANTU	BAS-CONGO	6	2010	0.38
2052_3	Typhi	2	2.5.1	no	yes	no	no	no	no	no	no	no	2052_3	931	KISANTU	BAS-CONGO	60	2009	0.25
2399_3	Typhi	2	2.5.1	no	yes	no	no	no	no	no	no	no	2399_3	3072	KISANTU	BAS-CONGO	12	2009	0.38
2531_3	Typhi	2	2.5.1	no	yes	no	no	no	no	no	no	no	2531_3	3204	KISANTU	BAS-CONGO	70	2010	0.38
2606_3	Typhi	2	2.5.1	no	yes	no	no	no	no	no	no	no	2606_3	3279	KISANTU	BAS-CONGO	5	2010	0.25
2072_3	Typhi	2	2.5.1	no	yes	no	no	no	no	no	no	no	2072_3	951	KISANTU	BAS-CONGO	19	2009	0.19
2557_3	Typhi	2	2.5.1	no	yes	no	no	no	no	no	no	no	2557_3	3230	KISANTU	BAS-CONGO	3	2010	0.38
10040_15	Typhi	2	2.5.1	no	yes	no	no	no	no	no	no	no	10040_15	9755	CS TAMBU-TSEKE/ DPS KWANGO	BANDUNDU	6	2015	.38

