# Supplementary figures and images for: Salmonella Typhi From Blood Cultures in the Democratic Republic of the Congo: A 10-Year Surveillance
Source: Clin Infect Dis. 2019 Mar 7;68(Suppl 2):S130–7. doi: 10.1093/cid/ciy1116 (PMC6405282; doi:10.1093/cid/ciy1116)

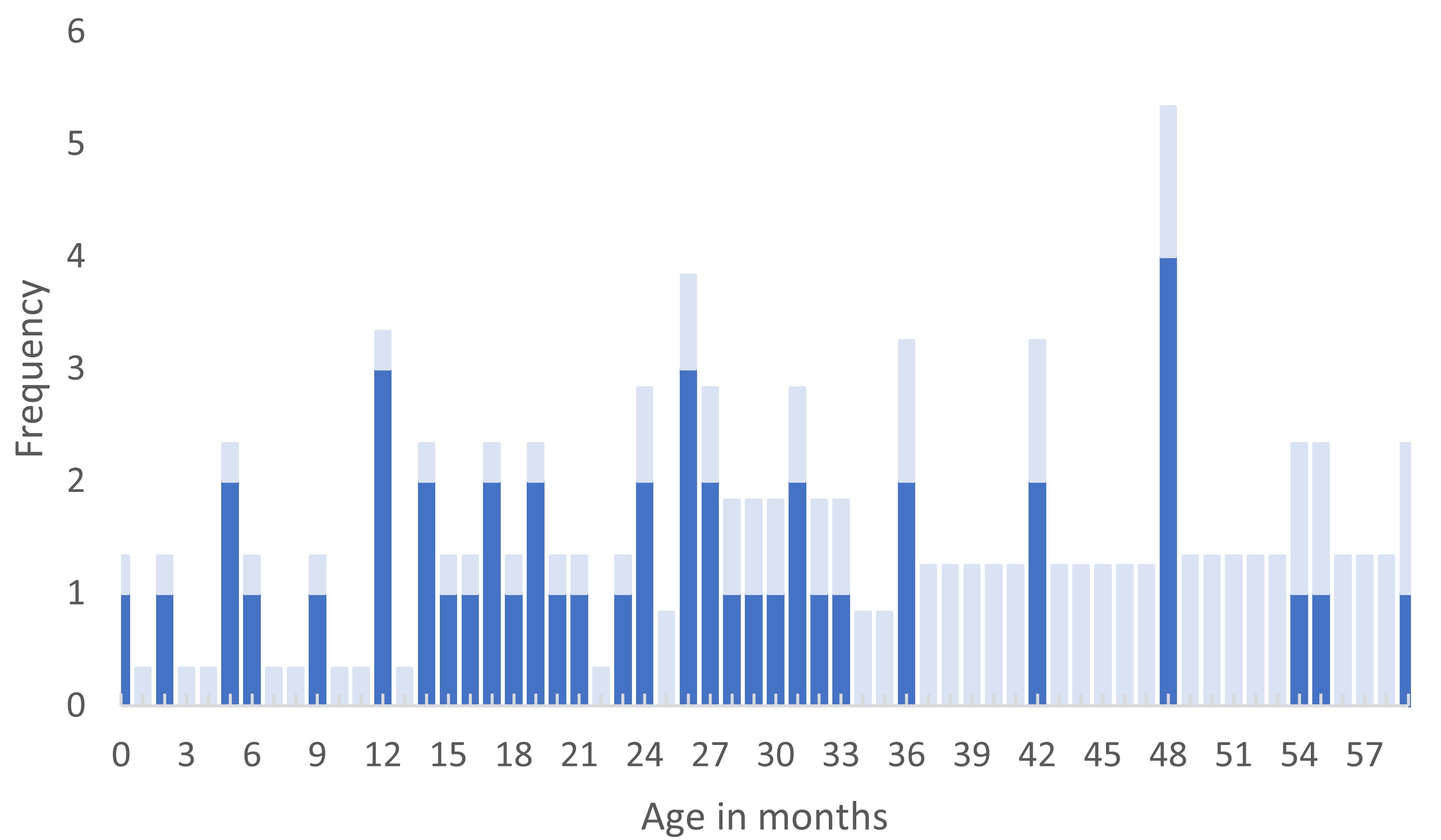

Supplement: ciy1116_suppl_Supplementary_Figure_1 [file ciy1116_suppl_supplementary_figure_1.jpeg]
