# Supplementary material for: Salmonella Typhi From Blood Cultures in the Democratic Republic of the Congo: A 10-Year Surveillance
Source: Clin Infect Dis. 2019 Mar 7;68(Suppl 2):S130–7. doi: 10.1093/cid/ciy1116 (PMC6405282; doi:10.1093/cid/ciy1116)
Supplement: ciy1116_suppl_Supplementary_Document_1 [file ciy1116_suppl_supplementary_document_1.docx]

**Supplementary Document 1. Work-flow, criteria and definitions used as part of the microbiological surveillance in the Democratic Republic of the Congo (DRC), 2007 – 2017.**

**Abbreviations:** CLSI: Clinical Laboratory Standards Institute, Wayne, Pennsylvania, U.S.; INRB = National Institute of Biomedical Research, Kinshasa, DRC; ITM = Institute of Tropical Medicine, Antwerp, Belgium: MoH: Ministry of Health, Sciensano: Belgian Institute for Health, Brussels, Belgium, hosting the National Reference Laboratory for *Salmonella* and *Shigella*.

**Note:** minor changes have been implemented over the years, according to practical issues or changes in reference documents -they are denoted by “*”. See text and cited references for full details.

**Study sites*:** see body text of the manuscript

The surveillance network of INRB was set up in Kinshasa in 2007 and later extended to sentinel hospitals in 4 Provinces of DRC that sampled blood cultures and either processed them on site (Kisantu, Kisangani) or shipped them to INRB (Bwamanda) for full analysis. In addition, blood cultures were added to the diagnostic tools of INRB in case of outbreak investigations initiated by MoH. Of note, the surveillance network is free-of-charge to the patient and the participating hospitals and health center. In 2015 the 11 former DRC-provinces were split in the actual 26 provinces.

**Indications for blood culture sampling as part of patient care:**

Indications for blood culture sampling (outside the neonatal period) comprise:

- a body temperature of ≥ 38.0°C or ≤ 35.5°C (axillary) or history of fever during the last 48 hours* and/or
- signs of severity such as hypotension, confusion, increased respiratory rate
- suspicion of severe localized infections (pneumonia, meningitis, complicated urinary tract infection, osteomyelitis and arthritis, severe skin and soft tissue infections, gynecological and abdominal infections/peritonitis)
- suspicion of other severe infection: sepsis, typhoid fever, and severe malaria

**Blood culture sampling:**

- Adults: 2 x 10 ml of blood sampled from separate venipunctures in aerobic (FA) BACT/ALERT bottles (bioMérieux, Marcy-L’Etoile, France)
- Children: 1 x 4 ml of blood sampled in pediatric (PF) BACT/ALERT bottles

**Blood culture processing used for patient care:**

- Incubation at 35°C for 7 consecutive days, visual check of chromogenic growth indicator daily
- Gram stain with subculture on appropriate culture media (Oxoid, Basingstoke, U.K. or Difco, Franklin Lakes, New Jersey, U.S.)
- Antibiotic susceptibility testing: disk diffusion according to CLSI guidelines*
- Storage of the bacterial isolates on Tryptone Soya Agar (Oxoid) and shipment to INRB and ITM for reference testing

**Data registration:**

- Basic demographic and clinical data as present on the laboratory request form were registered in an Excel database (Microsoft Corporation, Redmond, Washington, USA)
- Demographic data: age, gender, geographic origin
- Clinical data: presumptive clinical diagnosis with focus of infection, use of antibiotics prior to sampling, and, for the years 2007 – 2011, the case definitions of typhoid fever according to the MoH (non-complicated and complicated cases)*

**Children and Adults:**

- Child: a person ≤ 14 years old
- Adult: a person > 14 years old

**Contaminants, Pathogens, Bloodstream Infection episode:**

- Bacterial isolates grown from blood cultures were considered as contaminants or pathogens
- Skin and environmental contaminants: *Bacillus* spp., Coagulase-negative *Staphylococcus*, *Corynebacterium* spp., *Micrococcus* spp., *Cutibacterium* spp. (former *Propionibacterium* spp.*)* and *Lactobacillus* spp.* [1,2]
- Pathogens: all other bacterial species – note they were referred to as “clinically significant organisms” in previous reports and publications [3,4]
- Suspected Bloodstream infection episode (BSI): all blood cultures in a single patient sampled within a 2-weeks period according to the indications mentioned above. Blood cultures sampled > 2 weeks after the initial sample were considered as a separate (new) BSI episode.
- A BSI episode was considered as culture-confirmed when a pathogen grew in at least in one of the bottles.
- Polymicrobial bloodstream infection: a BSI episode was considered polymicrobial if different pathogens were isolated within maximum 48 hours apart from each other
- Recurrent pathogen: growth of the same isolate (species) in a next BSI episode, i.e. > 2 weeks after the growth of the initial pathogen

**Reference testing and INRB, ITM and Sciensano**

- Repeat identification and antibiotic susceptibility testing of pathogens, batch-testing.
- Phenotypic identification of *Salmonella* isolates: biochemical testing (aspects on Kligler Iron Agar (Oxoid; acid from glucose, gas, production of H_2_S), negative tests for urease, oxidase, β-galactosidase, indole production and citrate metabolization
- Serotyping with commercial antisera (Remel, Lenexa, Kansas and Sifin, Berlin, Germany). A 10% random set of Salmonella serotyping performed at ITM was submitted to Sciensano for quality control. Non-typhoidal *Salmonella* other than the Typhimurium and Enteritidis serotypes were serotyped by Sciensano according to the Kaufman-White scheme [5]
- Antimicrobial susceptibility testing: (i) disk diffusion (Neo-Sensitabs, Rosco, Taastrup, Denmark) according to CLSI guidelines* with (ii) assessing Minimal Inhibitory Concentrations (MIC-values) according to E-test macromethod (bioMérieux, Oxoid) for ciprofloxacin and azithromycin. Quality control was performed using American Type Culture Collection (ATCC) *Escherichia coli* 25922, *Staphylococcus aureus* 29213, and *Klebsiella pneumoniae* 700603. Surrogate disk diffusion test to predict non-susceptibility to ciprofloxacin was used with the antibiotics nalidixic acid and – since 2015 – pefloxacin.

**Definitions of Antimicrobial Resistance (AMR) profiles:**

- Multi-drug-resistant (MDR): resistant to the first-line oral antibiotics (amoxicillin, chloramphenicol, trimethoprim-sulfamethoxazole (TMP/SMX, co-trimoxazole)
- Although superseded, the term “Decreased Ciprofloxacin-Susceptibility” was maintained and used for ciprofloxacin MIC-values > 0.064 mg/L and < 1 mg/L, whereas “full ciprofloxacin resistance” was reserved for MIC-values ≥ 1 mg/ml [6,7].
- For azithromycin, isolates were considered resistant at MIC values > 16 mg/L [6]

**Statistical Analysis**

- Only the first isolate per BSI episode was considered for statistical work-up [8]
- Statistics: proportions were tested for significance using the χ2 test and differences in age distribution by the Wilcoxon Mann–Whitney nonparametric test and the median test. A p-value of <0.05 was considered significant.

**Reports about the surveillance:**

- April 2007 – January 2011: see Reference [3].
- January 2011 – December 2014: see Reference [4].
- January 2015 – October 2017: present study.

**References**

1. Isenberg H. Blood cultures: general detection and interpretation. In: Isenberg H, ed. Clinical microbiology procedures handbook. Vol 1. 2nd ed. Washington, DC: American Society for Microbiology Press, **2004**: 3.4.1.1–19.
2. Leber A.L. Chapter 3.4 Blood cultures 3.4.1.1 -3.4.2.6. In: Clinical Microbiology Procedures Handbook. AMS Press, **2016**. EBSCO publishing: ebook clinical collection https://health.ebsco.com/products/ebook-clinical-collection.
3. Lunguya O, Phoba MF, Mundeke SA, et al. The diagnosis of typhoid fever in the Democratic Republic of the Congo. Trans R Soc Trop Med Hyg **2012**; 106:348–55.
4. Kalonji LM, Post A, Phoba MF, et al. Invasive *Salmonella* infections at multiple surveillance sites in the Democratic Republic of the Congo, 2011–2014. Clin Infect Dis **2015**; 61:S346–53.
5. Grimont PA, Weill FX. Antigenic formulae of the *Salmonella* serovars. 9th ed. Paris, France: Institut Pasteur, 2007. Available at: <https://www.pasteur.fr/sites/default/files/veng_0.pdf>. Accessed 30 August 2018.
6. Clinical and Laboratory Standards Institute. Performance standards for antimicrobial susceptibility testing. 28^th^ edition **2018**; CLSI Document M100-S28.
7. Crump JA, Sjölund-Karlsson M, Gordon MA, Parry CM. Epidemiology, Clinical Presentation, Laboratory Diagnosis, Antimicrobial Resistance, and Antimicrobial Management of Invasive *Salmonella* Infections. Clin Microbiol Rev. **2015**; 28:901-37.
8. Clinical and Laboratory Standards Institute. Performance standards for antimicrobial susceptibility testing. 4^th^ edition **2014**; CLSI Document M39 A4.
