# Supplementary material for: Salmonella Typhi From Blood Cultures in the Democratic Republic of the Congo: A 10-Year Surveillance
Source: Clin Infect Dis. 2019 Mar 7;68(Suppl 2):S130–7. doi: 10.1093/cid/ciy1116 (PMC6405282; doi:10.1093/cid/ciy1116)
Supplement: ciy1116_suppl_Supplementary_Figure_Legend [file ciy1116_suppl_supplementary_figure_legend.docx]

**Supplementary Figure 1.** Age distribution in months of *Salmonella* Typhi bloodstream infection episodes for children aged less than five years. The dark blue bars represent the numbers of *Salmonella* Typhi for which the age in months was available (n = 46); the *Salmonella* Typhi bloodstream infection episodes for which only the age in years was available (n = 49) were allocated evenly across the months of that year and are represented by the light blue bars. Data were generated for a total of 519 out of 531 episodes for which data on age was recorded.
